# Supplementary material for: Integrated transcriptomic and proteomic analysis of Tritipyrum provides insights into the molecular basis of salt tolerance
Source: PeerJ. 2021 Dec 23;9:e12683. doi: 10.7717/peerj.12683 (PMC8710252; doi:10.7717/peerj.12683)
Supplement: Supplemental Information 1 [file peerj-09-12683-s001.doc]

**Table S1** The names and relative expression levels of differentially expressed wheat proteins

confirmed by qRT-PCR

| **Protein ID** | | | **Log_2_FC** | **Protein description** | | **Protein name** |
| --- | --- | --- | --- | --- | --- | --- |
| **Salt stress** | |  | | |  | |
| TraesCS7D01G510700.1 | | | -1.4626 | Phosphopantothenate--cysteine ligase 2-like | | PPCS2 |
| TraesCS7A01G463800.1 | | | -1.8003 | Xyloglucan endotransglucosylase/hydrolase protein 24 | | XTH24 |
| TraesCS6A01G169200.1 | | | -1.9113 | Glucomannan 4-beta-mannosyltransferase 1 | | CSLA01 |
| TraesCS2A01G548100.1 | | | -1.7391 | Probable polyamine oxidase 4 | | PAO4 |
| TraesCS2A01G352800.1 | | | -1.9551 | Endoglucanase 12 | | GH9B9 |
| TraesCS3B01G409300.1 | | | 2.9080 | Protein early responsive to dehydration 15-like | | ERD15 |
| TraesCS3A01G092800.1 | | | 2.7788 | Galactinol--sucrose galactosyltransferase | | RFS1 |
| TraesCS7A01G533000.1 | | | 2.2649 | Senescence/dehydration-associated protein At3g51250-like | | AT3G51250 |
| TraesCS1D01G369800.1 | | | 2.1085 | ABA-inducible protein PHV A1-like | | HVA1 |
|  |  |  | | |  | |
| **Recovery** | |  | | |  | |
| TraesCS6D01G295500.2 | | | -1.4910 | 24.1 kda heat shock protein, mitochondrial-like isoform X2 | | HSP24.1 |
| TraesCS3A01G023000.1 | | | -2.4635 | Glycosyltransferase family 61 protein | | AT3G18170 |
| TraesCS2B01G113200.1 | | | -1.5461 | Transcription factor TGA2.1 | | TGA2.1 |
| TraesCS1A01G251000.1 | | | -1.4248 | 1-(5-phosphoribosyl)-5-[(5-phosphoribosylamino) methylideneamino] imidazole-4-carboxamide isomerase | | APG10 |
| TraesCS7B01G232700.1 | | | 1.9105 | Spermidine synthase | | SPDS1 |
| TraesCS7A01G211200.1 | | | 2.1783 | Peroxidase 11 | | AT1G68850 |
