# Supplementary material for: Integrated transcriptomic and proteomic analysis of Tritipyrum provides insights into the molecular basis of salt tolerance
Source: PeerJ. 2021 Dec 23;9:e12683. doi: 10.7717/peerj.12683 (PMC8710252; doi:10.7717/peerj.12683)
Supplement: Supplemental Information 2 [file peerj-09-12683-s002.doc]

**Table S1** Common differentially expressed proteins of wheat “Y1805” and “Chinese Spring” under salt-stress conditions.

| **Protein ID** | **Y1805** | |  | **Chinese Spring** | |
| --- | --- | --- | --- | --- | --- |
|  | **log_2_FC** | **Q value** |  | **log_2_FC** | **Q value** |
| TraesCS1A01G295800.1 | 2.6392 | 0.0016 |  | 2.2167 | 0.0055 |
| TraesCS1D01G256800.1 | 1.4125 | 0.0353 |  | 1.4975 | 0.0164 |
| TraesCS2A01G154600.1 | -1.2153 | 0.0220 |  | -1.3895 | 0.0063 |
| TraesCS2A01G264900.1 | -1.2993 | 0.0024 |  | -1.0045 | 0.0128 |
| TraesCS2A01G292000.1 | 1.4703 | 0.0069 |  | 2.3869 | 0.0001 |
| TraesCS2A01G352800.1 | -1.9551 | 0.0080 |  | -1.7608 | 0.0131 |
| TraesCS2D01G377600.1 | 1.1991 | 0.0245 |  | 1.6253 | 0.0026 |
| TraesCS3A01G092800.1 | 2.7788 | 0.0002 |  | 3.2024 | 0 |
| TraesCS3B01G045500.1 | 1.3490 | 0.0100 |  | 1.7662 | 0.0013 |
| TraesCS3B01G409300.1 | 2.9080 | 0.0024 |  | 2.3946 | 0.0041 |
| TraesCS3D01G415100.1 | -1.6323 | 0.0179 |  | -2.1100 | 0.0055 |
| TraesCS4A01G114400.1 | -1.1262 | 0.0016 |  | -1.4895 | 0.0030 |
| TraesCS4A01G260200.1 | -1.4024 | 0.0153 |  | -1.4874 | 0.0075 |
| TraesCS4A01G275300.1 | 1.4882 | 0.0087 |  | 2.2316 | 0.0032 |
| TraesCS5A01G171700.1 | 1.3998 | 0.0447 |  | 1.589 | 0.0055 |
| TraesCS5A01G392000.1 | -1.7356 | 0.0007 |  | -1.3286 | 0.0055 |
| TraesCS6A01G169200.1 | -1.9113 | 0 |  | -1.7600 | 0.0001 |
| TraesCS7A01G204500.1 | -1.1792 | 0.0004 |  | -1.6208 | 0.0001 |
| TraesCS7A01G463800.1 | -1.8003 | 0 |  | -1.1557 | 0.0031 |
| TraesCS7A01G533000.1 | 2.2649 | 0.0164 |  | 2.7561 | 0.0042 |
| TraesCS7B01G188400.2 | -1.1615 | 0.0240 |  | -1.2309 | 0.0119 |
