# Supplementary material for: Integrated transcriptomic and proteomic analysis of Tritipyrum provides insights into the molecular basis of salt tolerance
Source: PeerJ. 2021 Dec 23;9:e12683. doi: 10.7717/peerj.12683 (PMC8710252; doi:10.7717/peerj.12683)
Supplement: Supplemental Information 3 [file peerj-09-12683-s003.doc]

**Table S2** Common differentially expressed proteins of wheat “Y1805” and “Chinese Spring” during the recovery process.

| **Protein ID** | **Y1805** | |  | **Chinese Spring** | |
| --- | --- | --- | --- | --- | --- |
|  | **log_2_FC** | **Q value** |  | **log_2_FC** | **Q value** |
| TraesCS1A01G266000.1 | 1.3271 | 0.0246 |  | 1.5707 | 0.0018 |
| TraesCS1A01G295800.1 | 3.3679 | 0.0003 |  | 2.8616 | 0.0002 |
| TraesCS1B01G096900.1 | 1.0809 | 0.0097 |  | 1.1389 | 0.0013 |
| TraesCS1B01G304800.1 | 3.6642 | 0.0030 |  | 4.1908 | 0.0002 |
| TraesCS1D01G214200.1 | -1.7302 | 0.0188 |  | -2.9377 | 0.0001 |
| TraesCS1D01G256800.1 | 1.3443 | 0.0277 |  | 1.6243 | 0.0018 |
| TraesCS1D01G369800.1 | 2.7734 | 0.0061 |  | 1.7202 | 0.0245 |
| TraesCS2A01G063500.1 | -1.4238 | 0.0273 |  | -1.0004 | 0.0325 |
| TraesCS2A01G183900.1 | 1.3925 | 0.0310 |  | 2.5188 | 0.0002 |
| TraesCS2A01G191600.2 | 1.3220 | 0.0006 |  | 1.0125 | 0.0010 |
| TraesCS2A01G292000.1 | 1.3863 | 0.0084 |  | 1.9999 | 0.0001 |
| TraesCS2A01G502100.1 | -1.6264 | 0.0057 |  | -2.4344 | 0.0001 |
| TraesCS2D01G230800.1 | -1.5572 | 0.0396 |  | -1.5201 | 0.0097 |
| TraesCS2D01G377600.1 | 1.3922 | 0.0069 |  | 1.6528 | 0.0004 |
| TraesCS4A01G266900.1 | 1.1259 | 0.0069 |  | 1.3571 | 0.0004 |
| TraesCS4A01G486000.1 | -1.4789 | 0.0471 |  | -1.4579 | 0.0117 |
| TraesCS4B01G347400.1 | 1.1560 | 0.0498 |  | -1.0191 | 0.0220 |
| TraesCS4B01G375200.1 | -1.7998 | 0.0033 |  | -1.4847 | 0.0020 |
| TraesCS5A01G014600.1 | -3.5581 | 0.0320 |  | -3.5094 | 0.0019 |
| TraesCS5A01G478800.1 | 3.3324 | 0.0013 |  | 2.2904 | 0.0025 |
| TraesCS5B01G355800.1 | 2.0848 | 0 |  | 1.2198 | 0.0002 |
| TraesCS6A01G169200.1 | -1.0807 | 0.0058 |  | -1.1228 | 0.0017 |
| TraesCS7A01G204500.1 | -1.3836 | 0.0002 |  | -1.5356 | 0.0002 |
| TraesCS7A01G211200.1 | 2.1783 | 0.0013 |  | 2.2243 | 0.0001 |
| TraesCS7A01G371600.1 | 1.2450 | 0.0282 |  | 1.4972 | 0.0019 |
| TraesCS7A01G539200.1 | -1.0094 | 0.0389 |  | -1.0064 | 0.0083 |
| TraesCS7B01G232700.1 | 1.9105 | 0.0144 |  | 1.0810 | 0.0466 |
| TraesCS7B01G307700.1 | 1.6865 | 0.0233 |  | 1.4466 | 0.0107 |
| TraesCS7B01G368100.1 | -1.8326 | 0.0043 |  | -1.2611 | 0.0072 |
