# Supplementary material for: Integrated transcriptomic and proteomic analysis of Tritipyrum provides insights into the molecular basis of salt tolerance
Source: PeerJ. 2021 Dec 23;9:e12683. doi: 10.7717/peerj.12683 (PMC8710252; doi:10.7717/peerj.12683)
Supplement: Supplemental Information 4 [file peerj-09-12683-s004.doc]

**Table S3** *Tritipyrum* “Y1805”-specific proteins during salt-stress and recovery conditions.

| **Protein ID** | **Salt stress** | |  | **Recovery** | |
| --- | --- | --- | --- | --- | --- |
|  | **log_2_FC** | **Q value** |  | **log_2_FC** | **Q value** |
| TraesCS1A01G295800.1 | 2.6392 | 0.0016 |  | 3.3679 | 0.0003 |
| TraesCS1B01G304800.1 | 2.5707 | 0.0402 |  | 3.6642 | 0.0030 |
| TraesCS1D01G256800.1 | 1.4125 | 0.0353 |  | 1.3443 | 0.0277 |
| TraesCS1D01G369800.1 | 2.1085 | 0.0436 |  | 2.7734 | 0.0061 |
| TraesCS2A01G292000.1 | 1.4703 | 0.0069 |  | 1.3863 | 0.0084 |
| TraesCS2A01G502100.1 | -1.2211 | 0.0447 |  | -1.6264 | 0.0057 |
| TraesCS2D01G377600.1 | 1.1991 | 0.0245 |  | 1.3922 | 0.0069 |
| TraesCS2D01G417100.2 | 2.2163 | 0.0179 |  | 1.7429 | 0.0144 |
| TraesCS3B01G045500.1 | 1.3490 | 0.0100 |  | 1.2088 | 0.0172 |
| TraesCS3D01G415100.1 | -1.6323 | 0.0179 |  | -1.3299 | 0.0354 |
| TraesCS4A01G275300.1 | 1.4882 | 0.0087 |  | 1.4926 | 0.0197 |
| TraesCS5A01G171700.1 | 1.3998 | 0.0447 |  | 1.6234 | 0.0157 |
| TraesCS6A01G169200.1 | -1.9113 | 0 |  | -1.0807 | 0.0058 |
| TraesCS7A01G204500.1 | -1.1792 | 0.0004 |  | -1.3836 | 0.0002 |
