# Supplementary material for: Integrated transcriptomic and proteomic analysis of Tritipyrum provides insights into the molecular basis of salt tolerance
Source: PeerJ. 2021 Dec 23;9:e12683. doi: 10.7717/peerj.12683 (PMC8710252; doi:10.7717/peerj.12683)
Supplement: Supplemental Information 6 [file peerj-09-12683-s006.doc]

**Table S6** The names and descriptions of the main proteins in the wheat PPI network.

| **Protein ID** | **Protein description** | **Protein name** |
| --- | --- | --- |
| **Salt stress** |  |  |
| TraesCS1A01G089500.1 | Ethylene-forming enzyme | EFE |
| TraesCS2A01G292000.1 | Arogenate dehydratase/prephenate dehydratase 6, chloroplastic | ADT6 |
| TraesCS2D01G377600.1 | Phenylalanine ammonia-lyase 1 | PAL1 |
| TraesCS7A01G204500.1 | Endoglucanase 17 | GH9B13 |
| TraesCS2A01G102600.1 | Probable cellulose synthase A catalytic subunit 8 [UDP-forming] | IRX1 |
| TraesCS1B01G136200.1 | Probable cellulose synthase A catalytic subunit 1 [UDP-forming] | CESA1 |
| TraesCS6A01G169200.1 | Glucomannan 4-beta-mannosyltransferase 1 | CSLA01 |
|  |  |  |
| **Recovery** |  |  |
| TraesCS1B01G077500.1 | Patatin-like protein 1 | PLP1 |
| TraesCS7A01G211200.1 | Peroxidase 11 | AT1G68850 |
| TraesCS2A01G191600.2 | Omega-hydroxypalmitate O-feruloyl transferase-like | RWP1 |
| TraesCS7A01G262000.1 | Peroxidase 55 | AT5G14130 |
| TraesCS4B01G352400.1 | Flavonoid O-methyltransferase-like protein Os11g0303600 | OMT1 |
| TraesCS2A01G502100.1 | Peroxidase 50 | AT4G37520 |
| TraesCS1B01G096300.1 | Peroxidase 5 | AT1G14550 |
| TraesCS2B01G612400.1 | Peroxidase 31-like | AT3G28200 |
| TraesCS5A01G234300.1 | 1-aminocyclopropane-1-carboxylate oxidase 1 | EFE |
| TraesCS7A01G204500.1 | Endoglucanase 17 | GH9B13 |
| TraesCS7A01G461500.1 | Solanesyl-diphosphate synthase 1, mitochondrial | SPS1 |
| TraesCS7A01G094100.1 | Nudix hydrolase 19, chloroplastic | NUDX19 |
| TraesCS2B01G379300.1 | UDP-glycosyltransferase 92A1 | AT5G12890 |
| TraesCS2D01G377600.1 | Phenylalanine ammonia-lyase | PAL1 |
| TraesCS2A01G292000.1 | Arogenate dehydratase/prephenate dehydratase 6, chloroplastic | ADT6 |
| TraesCS6A01G088200.2 | Ribose-phosphate pyrophosphokinase 1, chloroplastic | AT2G35390 |
| TraesCS1A01G251000.1 | 1-(5-phosphoribosyl)-5-[(5-phosphoribosylamino) methylideneamino] imidazole-4-carboxamide isomerase, chloroplastic | APG10 |
| TraesCS1B01G098300.2 | Peptide chain release factor APG3, chloroplastic | APG3 |
| TraesCS3A01G205300.1 | ATP-dependent zinc metalloprotease FTSH 5, mitochondrial | VAR1 |
| TraesCS4A01G266900.1 | Cytosolic glutamine synthetase | GSR2 |
| TraesCS1D01G214200.1 | Protein NRT1/ PTR FAMILY 6.3-like | NRT1.1 |
| TraesCS7B01G232700.1 | Spermidine synthase | SPDS1 |
| TraesCS7A01G539200.1 | Polyamine oxidase-like | PAO1 |
| TraesCS1B01G396200.1 | 1,2-dihydroxy-3-keto-5-methylthiopentene dioxygenase 4-like | ARD4 |
| TraesCS7A01G450600.1 | 50S ribosomal protein L24, chloroplastic | RPL24 |
| TraesCS4B01G347400.1 | Nascent polypeptide-associated complex subunit alpha-like protein 2 | NACA2 |
| TraesCS1D01G105500.1 | 60S ribosomal protein L18-2-like | RPL18 |
| TraesCS2A01G154400.1 | Dexh-box ATP-dependent RNA helicase dexh10 | HEN2 |
| TraesCS3A01G130200.1 | DEAD-box ATP-dependent RNA helicase 14-like isoform X2 | DRH1 |
| TraesCS7B01G371900.1 | Peptidyl-prolyl cis-trans isomerase CYP59-like | CYP59 |
| TraesCS6B01G254000.1 | PREDICTED: H/ACA ribonucleoprotein complex subunit 3-like protein | NOP10 |
| TraesCS5A01G267500.1 | Splicing factor u2af small subunit A | ATU2AF35A |
