# Supplementary material for: Integrated transcriptomic and proteomic analysis of Tritipyrum provides insights into the molecular basis of salt tolerance
Source: PeerJ. 2021 Dec 23;9:e12683. doi: 10.7717/peerj.12683 (PMC8710252; doi:10.7717/peerj.12683)
Supplement: Supplemental Information 7 [file peerj-09-12683-s007.doc]

**Table S7** Proteome-transcriptome-associated DEPs/DEGs in *Tritipyrum* ‘Y1805’ under salt-stress and recovery conditions.

| **Protein ID** | **Gene ID** | **Protein name** | **log_2_FC** | |
| --- | --- | --- | --- | --- |
|  |  |  | **Protein** | **Gene** |
| **Salt stress** | | | | |
| TraesCS1B01G096300.1 | TraesCS1B02G096300 | AT1G14550 | 1.0744 | 2.3374 |
| TraesCS1D01G256800.1 | TraesCS1D02G256800 | CXE15 | 1.4125 | 3.2664 |
| TraesCS1D01G369800.1 | TraesCS1D02G369800 | EMT12912 | 2.1085 | 5.9360 |
| TraesCS2A01G292000.1 | TraesCS2A02G292000 | ADT6 | 1.4703 | 2.4611 |
| TraesCS2D01G377600.1 | TraesCS2D02G377600 | PAL1 | 1.1991 | 1.2842 |
| TraesCS3A01G092800.1 | TraesCS3A02G092800 | RFS1 | 2.7788 | 1.8044 |
| TraesCS3A01G093900.1 | TraesCS3A02G093900 | EMT04243 | 1.3796 | 3.2853 |
| TraesCS7A01G533000.1 | TraesCS7A02G533000 | AT4G35985 | 2.2649 | 2.3706 |
| TraesCS1A01G295800.1 | TraesCS1A02G295800 | uncharacterized protein | 2.6392 | 9.3186 |
| TraesCS1B01G304800.1 | TraesCS1B02G304800 | uncharacterized protein | 2.5707 | 10.3932 |
| TraesCS2D01G417100.2 | TraesCS2D02G417100 | uncharacterized protein | 2.2163 | 7.6690 |
| TraesCS3B01G409300.1 | TraesCS3B02G409300 | ERD15 | 2.9080 | 1.0122 |
| TraesCS7A01G118300.1 | TraesCS7A02G118300 | AT2G33585 | 1.6873 | 2.9364 |
|  |  |  |  |  |
| **Recovery** | | | | |
| TraesCS1B01G307700.1 | TraesCS1B02G307700 | EMT03679 | 2.1310 | 6.7644 |
| TraesCS1D01G256800.1 | TraesCS1D02G256800 | CXE15 | 1.3443 | 1.5181 |
| TraesCS1D01G369800.1 | TraesCS1D02G369800 | HVA1 | 2.7734 | 3.6985 |
| TraesCS2A01G183900.1 | TraesCS2A02G183900 | BRU6 | 1.3925 | 1.6028 |
| TraesCS2A01G191600.2 | TraesCS2A02G191600 | RWP1 | 1.3220 | 1.1697 |
| TraesCS2A01G292000.1 | TraesCS2A02G292000 | ADT6 | 1.3863 | 1.3171 |
| TraesCS2D01G377600.1 | TraesCS2D02G377600 | PAL1 | 1.3922 | 1.4847 |
| TraesCS3A01G287400.1 | TraesCS3A02G287400 | SAT1 | 1.0807 | 1.1192 |
| TraesCS4A01G173800.1 | TraesCS4A02G173800 | Xip-R1 | 1.1897 | 2.2906 |
| TraesCS5A01G234300.1 | TraesCS5A02G234300 | EFE | 1.2115 | 1.1278 |
| TraesCS5B01G355800.1 | TraesCS5B02G355800 | EMT04678 | 2.0848 | 2.6149 |
| TraesCS7A01G211200.1 | TraesCS7A02G211200 | AT1G68850 | 2.1783 | 1.8185 |
| TraesCS7B01G273600.1 | TraesCS7B02G273600 | AT5G44410 | 1.2173 | 1.6264 |
| TraesCS1A01G295800.1 | TraesCS1A02G295800 | uncharacterized protein | 3.3679 | 6.0505 |
| TraesCS1B01G304800.1 | TraesCS1B02G304800 | uncharacterized protein | 3.6642 | 6.1560 |
| TraesCS2D01G417100.2 | TraesCS2D02G417100 | uncharacterized protein | 1.7429 | 4.1595 |
| TraesCS3A01G040800.1 | TraesCS3A02G040800 | BG | 1.0034 | 3.5218 |
| TraesCS3A01G348000.1 | TraesCS3A02G348000 | unnamed protein product | 2.1880 | 1.5345 |
| TraesCS3B01G045500.1 | TraesCS3B02G045500 | CML16 | 1.2088 | 1.8487 |
| TraesCS3D01G039300.1 | TraesCS3D02G039300 | BG | 1.1326 | 4.0370 |
| TraesCS3D01G039400.1 | TraesCS3D02G039400 | uncharacterized protein | 1.2282 | 2.4774 |
| TraesCS4D01G206500.1 | TraesCS4D02G206500 | uncharacterized protein | 1.4065 | 6.6856 |
| TraesCS5A01G478800.1 | TraesCS5A02G478800 | ADF2 | 3.3324 | 5.4122 |
| TraesCS7A01G371600.1 | TraesCS7A02G371600 | CHT1 | 1.2450 | 1.5368 |
| TraesCS7B01G307700.1 | TraesCS7B02G307700 | HSR201 | 1.6865 | 1.3771 |
