# Supplementary figures and images for: Integrated transcriptomic and proteomic analysis of Tritipyrum provides insights into the molecular basis of salt tolerance
Source: PeerJ. 2021 Dec 23;9:e12683. doi: 10.7717/peerj.12683 (PMC8710252; doi:10.7717/peerj.12683)

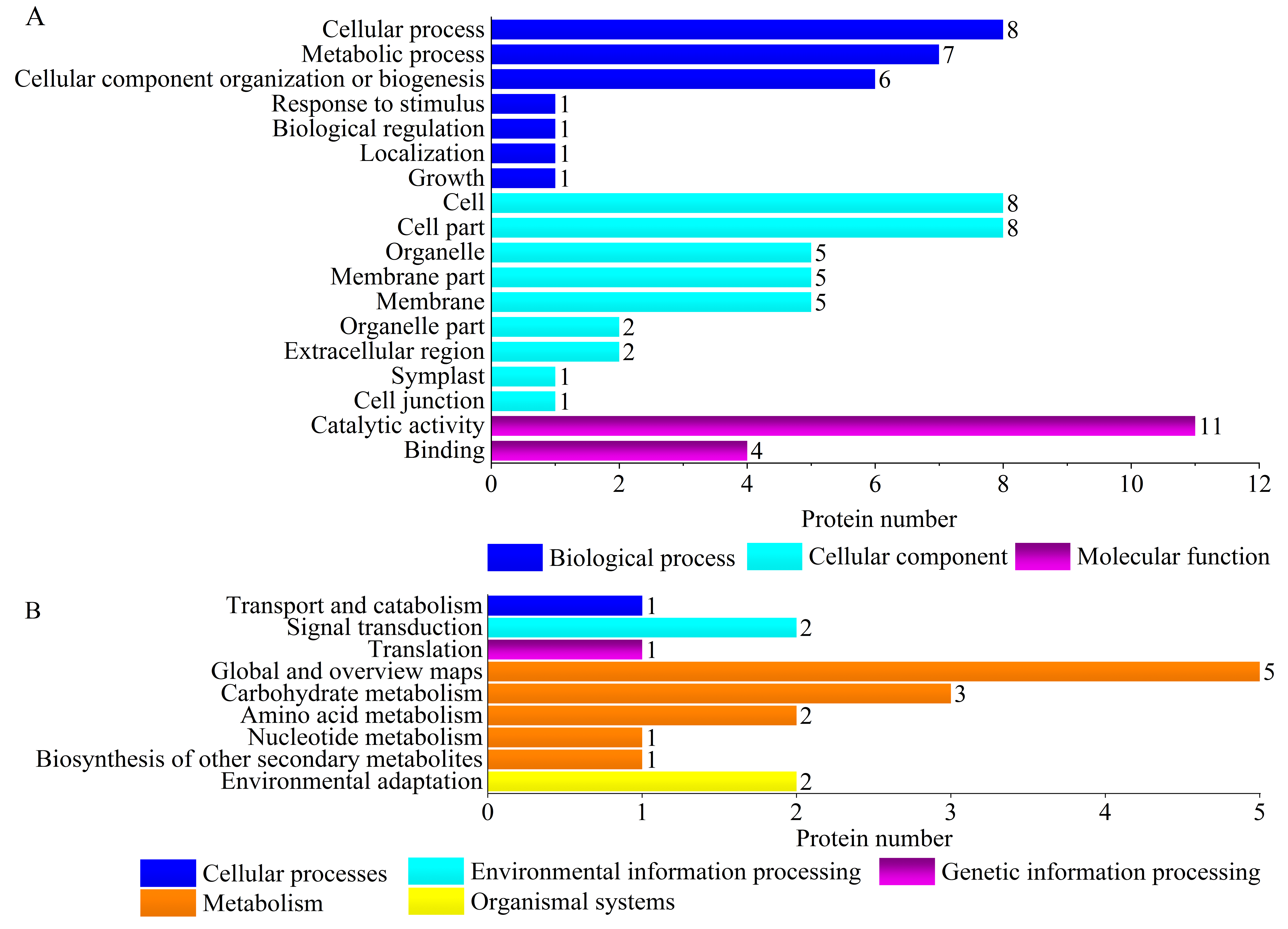

Supplement: Supplemental Information 8 [file peerj-09-12683-s008.png]

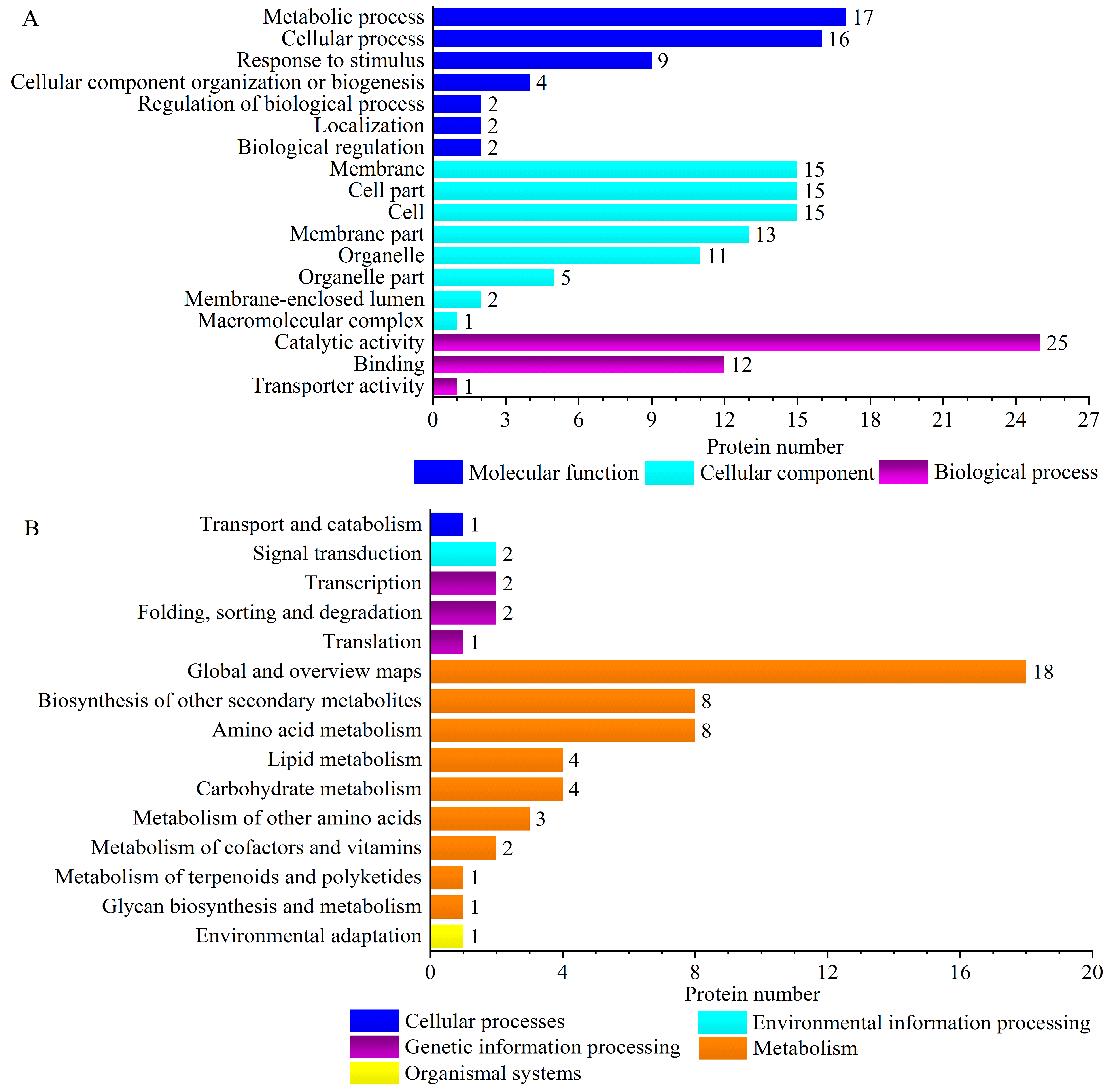

Supplement: Supplemental Information 9 [file peerj-09-12683-s009.png]
